# Detection of *Helicobacter pylori* Infection in Histopathological Gastric Biopsies Using Deep Learning Models

**DOI:** 10.3390/jimaging11070226

**Published:** 2025-07-07

**Authors:** Rafael Parra-Medina, Carlos Zambrano-Betancourt, Sergio Peña-Rojas, Lina Quintero-Ortiz, Maria Victoria Caro, Ivan Romero, Javier Hernan Gil-Gómez, John Jaime Sprockel, Sandra Cancino, Andres Mosquera-Zamudio

**Affiliations:** 1 Departamento de Patología, Fundación Universitaria de Ciencias de la Salud (FUCS), Bogotá 111411, Colombia; lmquintero@fucsalud.edu.co (L.Q.-O.); mvcaro@fucsalud.edu.co (M.V.C.); iaromero1@fucsalud.edu.co (I.R.); 2 Instituto de Investigación, Fundación Universitaria de Ciencias de la Salud (FUCS), Bogotá 111411, Colombia; cazambranob@unbosque.edu.co (C.Z.-B.); jgilg@unbosque.edu.co (J.H.G.-G.); jjsprockel@fucsalud.edu.co (J.J.S.); 3 Departamento de Patología, Instituto Nacional de Cancerología (INC), Bogotá 111511, Colombia; scancino@cancer.gov.co; 4 Maestría en Estadística Aplicada y Ciencia de Datos, Universidad El Bosque, Bogotá 111321, Colombia; spenar@unbosque.edu.co; 5 Departamento de Ingeniería Eléctrica y Electrónica, Universidad del Norte, Barranquilla 080003, Colombia; 6 Laboratorio de Patología, Clínica Colsanitas, Bogotá 111711, Colombia; andres.mosquera@keralty.co

**Keywords:** *Helicobacter pylori*, whole-slide images, deep learning, deep convolutional neural network

## Abstract

Traditionally, *Helicobacter pylori* (HP) gastritis has been diagnosed by pathologists through the examination of gastric biopsies using optical microscopy with standard hematoxylin and eosin (H&E) staining. However, with the adoption of digital pathology, the identification of HP faces certain limitations, particularly due to insufficient resolution in some scanned images. Moreover, interobserver variability has been well documented in the traditional diagnostic approach, which may further complicate consistent interpretation. In this context, deep convolutional neural network (DCNN) models are showing promising results in the automated detection of this infection in whole-slide images (WSIs). The aim of the present article is to detect the presence of *HP* infection from our own institutional dataset of histopathological gastric biopsy samples using different pretrained and recognized DCNN and AutoML approaches. The dataset comprises 100 H&E-stained WSIs of gastric biopsies. HP infection was confirmed previously using immunohistochemical confirmation. A total of 45,795 patches were selected for model development. InceptionV3, Resnet50, and VGG16 achieved AUC (area under the curve) values of 1. However, InceptionV3 showed superior metrics such as accuracy (97%), recall (100%), F1 score (97%), and MCC (93%). BoostedNet and AutoKeras achieved accuracy, precision, recall, specificity, and F1 scores less than 85%. The InceptionV3 model was used for external validation, and the predictions across all patches yielded a global accuracy of 78%. In conclusion, DCNN models showed stronger potential for diagnosing HP in gastric biopsies compared with the auto ML approach. However, due to variability across pathology applications, no single model is universally optimal. A problem-specific approach is essential. With growing WSI adoption, DL can improve diagnostic accuracy, reduce variability, and streamline pathology workflows using automation.

## 1. Introduction

*Helicobacter pylori* (HP) is a microaerophilic, Gram-negative bacillus capable of colonizing the gastric mucosa. It infects more than half of the world’s population, making it the most common bacterial infection [1]. The prevalence of HP varies greatly, ranging from 20% to 80% within populations. It is particularly high in developing countries, where its occurrence is closely linked to socioeconomic status and hygiene conditions. Although the exact route of transmission remains unclear, it is generally believed that the bacterium is acquired during childhood via the fecal–oral route, with intrafamilial spread being common. Contaminated water or vegetables have also been implicated [1].

In 1994, the World Health Organization, through the International Agency for Research on Cancer (IARC), classified HP as a Group 1 carcinogen. Infection with this microorganism is the principal risk factor for the development of gastric cancer, exhibiting a significant positive association and a relative risk of 3.8 [2]. Most infected individuals remain asymptomatic; however, 10 to 20% may progress to conditions such as atrophic gastritis, peptic ulcer disease, gastric adenocarcinoma, or mucosa-associated lymphoid tissue (MALT) lymphoma, with less than 3% developing other forms of gastric cancer. The progression of these pathologies is influenced by the host’s genetic predisposition and immune response.

The diagnosis of HP is typically performed by pathologists using optical microscopy with standard H&E (hematoxylin and eosin) staining. Definitive identification of HP infection relies on visualizing the minute bacilli, which are approximately 2–4 μm in length and 0.5–1 μm in width [3]. The bacteria are mostly located at the borders of the tissue samples. However, the small size of these organisms within tissue sections often renders detection challenging. Although histologic clues—such as a superficial, band-like inflammatory infiltrate rich in plasma cells in the antral mucosa—may support the diagnosis, these features are not invariably present, and mucus debris can further obscure the bacteria [4]. Consequently, ancillary techniques such as Giemsa or Warthin–Starry stains and immunohistochemical (IHC) methods are sometimes employed to enhance diagnostic accuracy, though their higher cost and extended turnaround time often preclude routine use in many clinical settings [5]. Moreover, the evaluation is time-consuming and highly dependent on the observer’s expertise, as studies have reported only moderate to good interobserver agreement among pathologists diagnosing HP gastritis [6].

Deep convolutional neural network (DCNN) models are increasingly being applied in pathology practice for a wide variety of tasks such as image pattern recognition for predicting disease diagnosis, prognosis, and therapeutics [7]. The advancement of computational hardware in recent years, especially tensor processing units (TPUs) and graphical processing units (GPUs), has allowed the improvement and broader application of DCNN [8]. In the last few years, several studies have proposed different approaches for the detection of HP in histopathological images. The aim of our study was to evaluate the performance of well-established DCNN models and AutoML (Automated Machine Learning) in the classification of HP in whole-slide images (WSIs), using HP IHC as the reference standard.

The main contributions of this work are as follows: (1) the development of a curated dataset of 100 H&E-stained gastric biopsies with IHC-confirmed HP annotations; (2) a comparative evaluation of multiple DCNN and AutoML models, highlighting the superior performance of InceptionV3; and (3) the use of Grad-CAM visualizations to enhance interpretability and biological relevance of the model predictions.

The remainder of this paper is organized as follows: Section 2 describes the dataset, annotations, model architectures, and evaluation methodology. Section 3 presents the experimental results and external validation. Section 4 discusses the implications and limitations of our findings, summarizes the relevant literature, and reviews previous approaches to HP detection using digital pathology (DP). Section 5 concludes the study and outlines future directions.

### Related Work

Gonçalves, et al. [9] reported high performance with these models. They used a curated public dataset (DeepHP (H&E slides captured by a microscope at a magnification of 20×)) consisting of 13,921 images derived from 19 histopathological WSIs, including 14 gastric mucosae without morphological changes (9926 negative HP samples images) and five with HP infection (3995 positive sample images). Their best results were obtained using VGG16 (AUC 0.998) as well as InceptionV3 and ResNet50 (AUC 0.994). The first studies developed by Klein, et al. [10] trained a VGG-style neural network on Giemsa- and H&E-stained slides. Their model achieved perfect sensitivity (1.0) but relatively low specificity (0.66) in classifying Giemsa-stained samples. Liscia DS, et al. [11] similarly used a VGG-based model on silver-stained samples, obtaining higher specificity (0.87) and sensitivity (0.89), but a precision of only 77%, due to the localized distribution of HP colonies and the large size of WSIs. In addition, Martin, et al. [12] also achieved strong performance using HALO-AI (fully convolutional VGG network) across two phases (phase 1: 70 HP biopsies and phase 2: 28 HP biopsies). They found a 0.96 sensitivity, 1.0 specificity, 0.99 accuracy, and 0.98 F1 score.

Ibrahim AU, et al. [13]. evaluated different DL architectures using a 5-fold cross-validation approach. They found ResNet-101 to be the best performer, followed by DenseNet- 201 with AUC values of 0.9417 and 0.9383, respectively. They used 204 images of H&E-stained slides (103 images of HP positive and 101 images of HP negative) in 50 cases. The images were captured at 400× magnification using a light microscope with a digital camera system. Similarly, Zhou S, et al. [14] used a dataset of 245 patients (160 HP-positive and 151 HP-negative biopsies) with diagnostic confirmation by H&E and IHC. They employed an ensemble model combining output probabilities from three ResNet-18 and three DenseNet-121 models. This ensemble achieved a sensitivity of 0.87, a specificity of 0.92, and F1 score of 0.89 for the diagnosis of WSIs. Additionally, they assessed the potential of their model as a support tool for pathologists during visual inspection of WSIs. Their results indicated that patch-level probability guidance notably enhanced diagnostic accuracy for HP positive samples but introduced greater diagnostic uncertainty when evaluating HP-negative samples.

Recently, Krishna S, et al. [15] tested several CNN models (VGG, VGG + XGBoost, Inception V3, Inceptionv3 + XGBoost, ResNet50, ResNet50 + XGBoost, 6-layer CNN model, BoostedNet) using two publicly available datasets (DeepHP) and Giemsa-stained gastric histopathological images. The BoostedNet model consists of two primary components, a CNN feature extractor and an XGBoost Classifier. They demonstrated that BoostedNet had superior performance even with a six-layer CNN model with data augmentation, achieving an accuracy of 98.41%, a precision of 98.56%, a recall (sensitivity) of 98.07%, an F1 score of 98.31%, a specificity of 98.71%, and a MCC of 96.82%.

Another study that demonstrated strong performance was conducted by Lin et al. [16] who developed a two-tiered deep-learning model. The primary model, trained using slide-level labels from 885 WSIs, demonstrated strong diagnostic performance, achieving an AUC of 0.974, sensitivity of 93.3%, and specificity of 90.1%, surpassing pathologist specificity (84.2%). Additionally, an auxiliary model trained on localized areas within positive slides showed an average precision of 0.58 for pinpointing regions infected by HP. Moreover, a recent article by Cano, et al. [17] using a database of 245 WSIs with only 163 positive patches presented a DL system for the diagnosis of HP on IHC-stained gastric mucosa based on trained autoencoders. This dataset of positive annotations was used to train baseline thresholding and an SVM using the features of a pretrained RedNet-18 and ViT models. A 10-fold cross-validation showed a performance of 91% accuracy, 86% sensitivity, 96% specificity, and 0.97 AUC in the diagnosis of HP.

## 2. Materials and Methods

**Figure 1:**
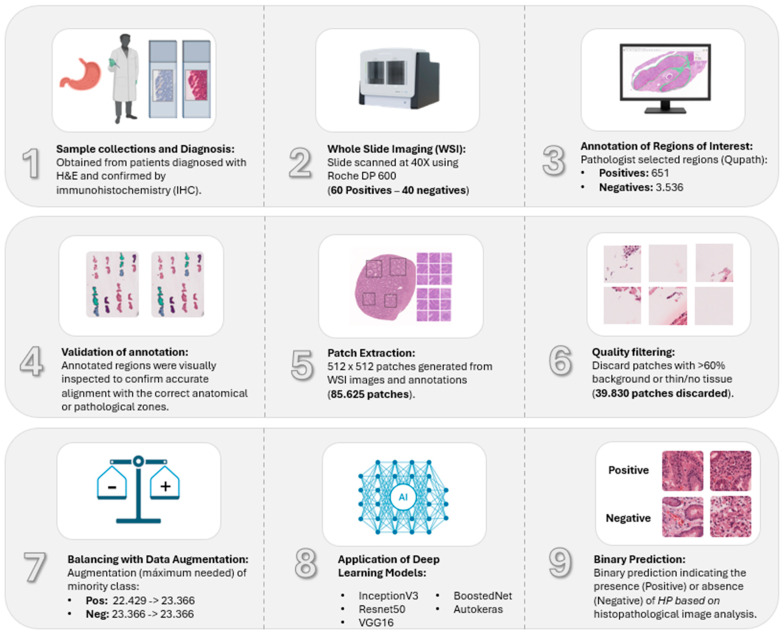
End-to-end histopathology image classification pipeline.

This section elucidates the proffered decision-support model for the detection of HP in WSIs. Figure 1 presents a flow diagram outlining the proposed approach.

### 2.1. Dataset and Annotations

In total, 20 patients from the Urkinina 5000 project were included [18]. Each patient had two biopsies from the antrum, two from the body, and one from the incisura, according to the Sydney system biopsy protocol. A total of 100 H&E-stained, formalin-fixed paraffin-embedded (FFPE) slides of gastric tissue biopsies were collected. Ethical approval was obtained from the institution’s ethics committee (internal record: 32-2022). The research team adhered to the Declaration of Helsinki and national regulations on research ethics (Resolution 8430 of 1993).

The samples were divided into 60 HP-positive biopsies (12 patients) and 40 HP-negative (8 patients) biopsies. The diagnosis was made using H&E staining and confirmed with HP immunohistochemical (IHC) evaluation. All slides were scanned at 40× magnification on VENTANA DP600 slide scanner (Roche Diagnostics, Basel, Switzerland).

All images were annotated using QuPath [19] by junior pathologists and reviewed by two senior pathologists from our institution. We labelled the H&E WSIs into HP-positive (*n* = 651) and HP-negative (*n* = 3536) groups. Annotations of HP-positive H&E slides were made in the same regions as the corresponding IHC stained areas, which included HP and glandular and inflammatory cells. For HP-negative slides, similar areas were annotated on H&E, including regions with intestinal metaplasia.

### 2.2. Immunohistochemical Staining

Briefly, 4 μm tissue sections mounted on Superfrost Plus slides (Thermo Scientific, Saint-Herblain, France) were dried overnight at 37 °C before processing. Immunohistochemical staining was performed with a Ventana BenchMark ULTRA autostainer (Ventana, Tucson, AZ, USA). The tissue sections were dewaxed and rehydrated. Antigen retrieval was performed by incubating slides. The slides were incubated with a rabbit anti-Helicobacter pylorus (SP48) (Ventana, USA). The slides were then incubated with a polymer-HRP reagent (OptiView DAB IHC detection kit, Ventana). Peroxidase activity was visualized using DAB solution and the slides were counterstained with hematoxylin.

### 2.3. Image Pre-Processing

To ensure annotation quality, a visualization pipeline was developed to overlay polygonal regions from GeoJSON files onto the WSIs. The images were loaded using OpenSlide [20]. Annotations were extracted, geometrically transformed with the Shapely library [21], and visualized as color-coded polygons using Matplotlib [22]. This combination provided immediate feedback on annotation accuracy, spatial alignment, and semantic consistency, ensuring high-quality, interpretable spatial data for downstream tasks.

**Figure 2:**
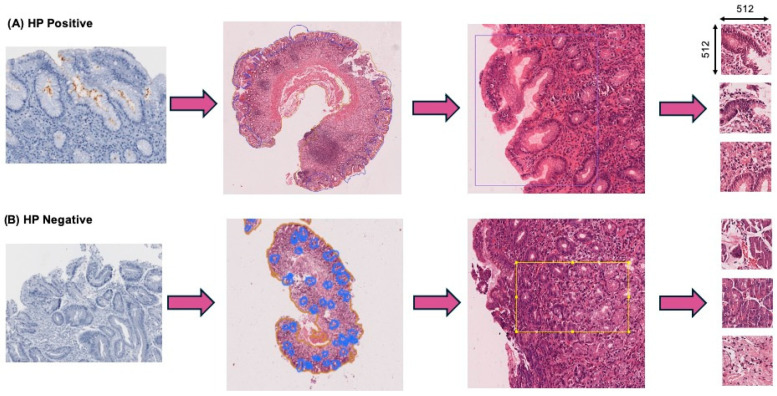
Data annotations and patch processing.

To construct a clinically meaningful training dataset, the original WSIs were segmented into 512 × 512-pixel patches at the highest resolution (level 0). Pathologist annotations—encoded in GeoJSON format and representing infected and non-infected regions—were rasterized using Rasterio and Shapely. Manual annotation of HP-positive and HP-negative regions was performed in the foveolar and glandular zones of gastric biopsies to identify areas with and without *HP* infection. This process yielded a patch-level dataset with binary labels (presence vs. absence of *HP*), grounded in expert-defined regions of interest (Figure 2).

### 2.4. Dataset Filtering and Quality Assurance

To exclude non-informative patches from WSIs, an automated RGB-based background filter (range: 180–245 across channels) was applied, removing patches with more than 60% background pixels. Subsequently, spatial validation ensured alignment of the remaining patches with annotation polygons. Specifically, patches from HP-positive WSIs were retained only if they intersected infection-marked regions, while patches from HP-negative WSIs required overlap with annotated, infection-free glandular areas.

### 2.5. Data Augmentation and Class Balancing

Image augmentation techniques are primarily divided into two approaches: traditional methods, which apply geometric transformations or color adjustments, and those based on deep learning algorithms, such as generative adversarial networks. These strategies are run exclusively on the training set during the model fitting phase, while the test data remain unchanged to preserve the validity of the evaluation [23,24].

To correct class imbalance and strengthen model robustness, we adopt traditional data augmentation techniques. Specifically, we targeted augmentation solely to the minority class using rotations up to 15° (rotation_range = 15), horizontal flips (horizontal_flip = True), 10% translations in width and height (width_shift_range = 0.1, height_shift_range = 0.1), moderate zooming (zoom_range = 0.1), and edge filling with the nearest mode (fill_mode = ‘nearest’). These transformations introduced realistic histological variations without altering class identity and were applied until class parity was achieved, ensuring the inclusion of all original samples.

### 2.6. Deep Convolutional Neural Network Models

To evaluate performance in the binary classification task (HP positive vs. negative), we applied both traditional DCNNs and AutoML approaches. Established DCNN models such as VGG16, ResNet50, InceptionV3, and BoostedNet were trained and tested on our balanced dataset of gastric biopsy image patches. In addition, we evaluated an AutoML-based approach using AutoKeras to assess its performance under the same experimental conditions. For all models, the dataset was randomly split into 80% training and 20% testing subsets to ensure robust model validation.

### 2.7. Validation and Interpretability with GradCAM

The model with the best external validation performance was selected for further interpretability analysis. The same annotation and image preprocessing methodology was applied to a new case consisting of five gastric biopsies from a single patient completely independent from training and testing dataset. Each image was resized to 299 × 299 pixels and passed through the trained model to generate class predictions. Grad-CAM was applied to produce heatmaps, highlighting the image regions that most influenced the model’s decision. These heatmaps were overlaid onto the original images and reviewed by an expert pathologist. The highlighted regions frequently corresponded to morphologically relevant areas, supporting the biological plausibility and interpretability of the predictions.

### 2.8. Experimental Design

We develop our applications in Python 3.12.7, with the Application Programming Interface (API) Keras (version 2.15.0), working with TensorFlow (version 2.15.0). The training approach is implemented using computer hardware from Fundación Universitaria de Ciencias de la Salud (FUCS) with 64 GB of DDR5-4800 ECC REG RAM, an NVIDIA RTX A6000 graphics processor with 48 GB of GDDR6 memory, and an Intel Xeon W9-3495X CPU with 56 performance cores running at 1.9 GHz (up to 4.8 GHz turbo). The system also includes a 4 TB HP Z Turbo PCIe 4 × 4 SSD, providing high-speed data access suitable for handling large image datasets.

### 2.9. Statistical Analysis

Data processing, model training, and evaluation were performed using Python 3.12.7 and associated libraries, including OpenCV (4.9.0), NumPy (1.26.4), SciPy (1.13.1), Matplotlib (3.8.4), Scikit-learn (1.4.2), Torch (PyTorch 2.2.2), and TensorFlow (2.15.0). Model selection was based on a comprehensive evaluation combining conventional performance metrics—accuracy, precision, recall (sensitivity), F1 score, specificity, and area under the ROC curve (AUC)—together with error distribution analysis using confusion matrices and the Matthews correlation coefficient (MCC).

These terms, including TP (true positive), TN (true negative), FP (false positive), and FN (false negative), refer to cases correctly classified as positive, correctly classified as negative, misclassified as positive, and misclassified as negative, respectively.

(1) $$\mathrm{Accuracy}=\frac{\mathrm{TP}+\mathrm{TN}}{\mathrm{TP}+\mathrm{TN}+\mathrm{FP}+\mathrm{FN}}$$

(2) $$\mathrm{Precision}=\frac{\mathrm{TP}}{\mathrm{TP}+\mathrm{FP}}$$

(3) $$\mathrm{Recall}=\frac{\mathrm{TP}}{\mathrm{TP}+\mathrm{FN}}$$

These metrics evaluate fundamental aspects of performance: accuracy quantifies the overall percentage of correct predictions; precision minimizes false positives, reducing the risk of unnecessary interventions in healthy samples; and recall ensures complete detection of pathological cases, avoiding false negatives. Furthermore, AUC complements these indicators by measuring the model’s discriminatory capacity across all possible thresholds, independent of class balance.

(4) $$\mathrm{Specificity}=\frac{\mathrm{TN}}{\mathrm{TP}+\mathrm{TN}}$$

(5) $$F1-\mathrm{Score}=\frac{2\mathrm{TP}}{2\mathrm{TP}+\mathrm{FP}+\mathrm{FN}}$$

(6) $$\mathrm{MCC}=\frac{\left(\mathrm{TP}\times \mathrm{TN})-(\mathrm{FP}\times \mathrm{FN}\right)}{\sqrt{\left(\mathrm{TP}+\mathrm{FP})(\mathrm{TP}+\mathrm{FN})(\mathrm{TN}+\mathrm{FP})(\mathrm{TN}+\mathrm{FN}\right)}}$$

Specificity represents the proportion of true correctly classified negatives, which allows a reduction in the incidence of false positives and ensure that non-pathological cases are not erroneously labeled. The F1 score, by calculating the harmonic mean between precision and sensitivity, provides a balanced metric of the model performance, particularly useful in contexts with unbalanced classes. For its part, the Matthews correlation coefficient (MCC) integrates all categories of classification analysis (TP, TN, FP, and FN) in a single value, offering a robust and balanced measure of global performance, especially suitable for clinical environments where diagnostic precision is critical.

## 3. Results

### 3.1. Dataset Curation and Augmentation Strategy

From an initial dataset of 85,625 image patches (each size 512 × 512 pixels) extracted from annotated HP positive and negative WSIs, a total of 45,795 high-quality patches remained after the quality control filtering process previously described, comprising 22,429 positive and 23,366 negative samples.

To correct class imbalance without discarding real data, we applied data augmentation on the positive class using geometric transformations (rotation, zoom, horizontal flip, etc.), resulting in a balanced dataset of 46,732 patches, with 23,366 per class. This balanced dataset was then used to train and evaluate the selected models (Inception V3, Resnet50, VGG16, BoostedNet, and AutoKeras).

To train and evaluate the model, an 80/20 split was performed based on biopsies, ensuring patient-level separation between phases (Table 1). To prevent cross-phase contamination, no individual provided biopsies for more than one study phase

### 3.2. Model Performance

Using the full dataset, a split was performed for training (37,385 patches) and testing (9.347 patches), which were used to evaluate the DL models. An overview of the performance metrics is presented in Table 2 (training) and Table 3 (testing). InceptionV3 achieved superior results in testing metrics such as accuracy (97%), recall (100%), F1 score (97%), and MCC (93%). Regarding precision and specificity, InceptionV3, VGG16 and ResNet50 had the same performance with 94% for both metrics. BoostedNet and AutoKeras had accuracy, precision, recall, specificity and F1 scores less than 85% with a MCC of 68% for BoostedNet and 64% for AutoKeras.

**Figure 3:**
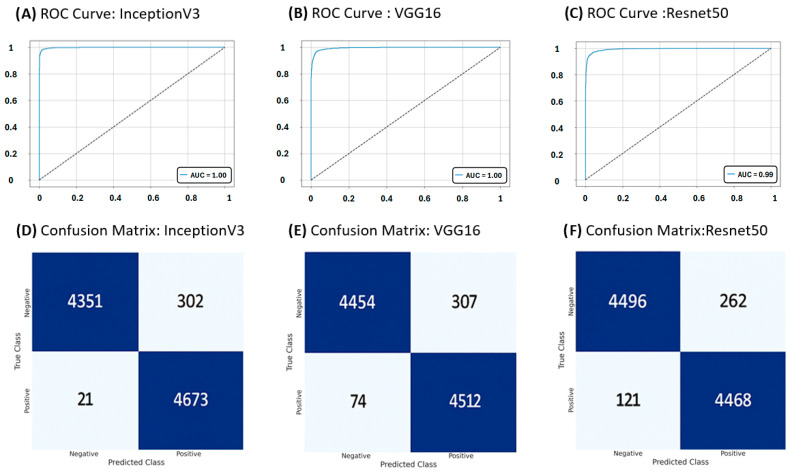
The ROC curves and confusion matrices. ROC of (**A**) InceptionV3, (**B**) VGG16, (**C**) Resnet50. Confusion matrices of (**D**) InceptionV3. (**E**) VGG16, (**F**) Resnet50.

The ROC curves and confusion matrices for the classification of histopathological images using DL models with better performance are presented in Figure 3A–C. InceptionV3, VGG16, and Resnet50 showed an AUC of 1. However, in the analysis of the confusion matrix, VGG16 and Resnet50 revealed high false negatives and false positives compared with InceptionV3 (Figure 3D–F).

### 3.3. External Validation

**Figure 4:**
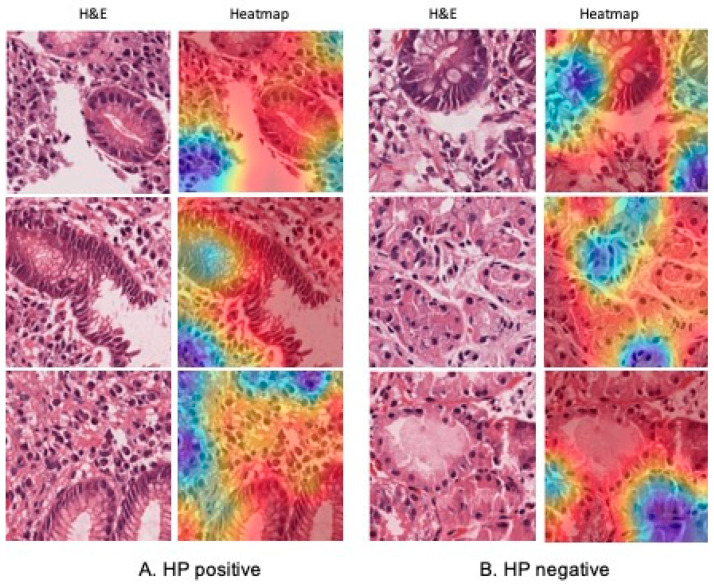
Grad CAM visualization of the InceptionV3 model in external validation with adequate prediction.

For external validation, a total of 3306 image patches (512 × 512 pixels) were used, including 179 HP positives and 3127 negatives. Using the InceptionV3 model, predictions across all patches yielded a global accuracy of 78%. While the model demonstrated high precision for negative cases (95%) and a specificity of 82%, sensitivity for positive patches was lower (25%), reflecting the morphological complexity of infected regions, which may also contain healthy tissue. Grad-CAM visualizations confirmed that the model focused on biologically plausible areas, such as glandular surfaces where HP resides, but also highlighted the most relevant regions driving each prediction. These visual cues enhanced the interpretability and transparency of the model’s decision-making process (Figure 4).

## 4. Discussion

In the present article, we used different DL models previously reported in the scientific literature and observed that InceptionV3 demonstrated superior performance on both training and test datasets, with no signs of overfitting due to an AUC of 1 and better metrics in testing with an accuracy of 97%, precision of 94%, recall of 100%, and F1 score of 97% (Table 3). To our knowledge, the InceptionV3 model is useful in big-data scenarios where huge amounts of data need to be processed at reasonable cost or scenarios where memory or computational capacity is inherently limited.

In the external validation experiment, the global accuracy for detecting *HP* was 78%. However, sensitivity for positive cases remained low, with a recall of 25% and precision of 7%, likely due to the morphological variability within annotated regions (Figure 2). Patches labeled positive often include glands with HP presence and band-like inflammatory infiltrate rich in plasma cells. To better understand the model’s predictions, Grad-CAM was applied, highlighting the regions of each patch that contributed to the classification. These activation maps, reviewed by an expert pathologist, confirmed that the model frequently focused on histologically relevant regions, thereby supporting the biological plausibility of the model’s decision-making process.

However, following external validation, a decrease in model sensitivity was observed, highlighting a potential lack of generalizability. This decrease can be attributed to multiple technical factors, such as variations in tissue preparation, staining protocols, and slide scanning procedures, which can significantly influence model training, especially when limited to data from a single center [25].

Moreover, the small training set size may not adequately capture the clinical and histological diversity required for robust performance, limiting the model’s generalization capabilities. Several studies have pointed out that many current computational pathology solutions still lack robustness against heterogeneity in tissue types, processing protocols, and scanning, highlighting the urgent need to develop more generalizable approaches suitable for reliable multicenter applications [26]. Also, another potential factor contributing to the reduced sensitivity may be inconsistency in the ground truth labeling, particularly in patches located at the boundaries of regions colonized by HP. These edge patches may have been labeled as negative despite containing tissue structures or features associated with the presence of the bacteria, potentially leading to misclassification during model training and evaluation.

Different approaches have been used in the last years to detect the presence of HP. The main works are based on different DL methods for the classification of cropped patches extracted from HP-positive or -negative samples using a stain or combined with H&E, ancillary techniques such as Giemsa or Warthin–Starry stains, and IHC. In the scientific literature, several articles showed high performance of AUC using models based on DCCN, such as Inception V3, Resnet50, VGG16, and MobileNet-V2; hybrid models, such as BoostedNet (CNN + XGBoost); or enhanced streaming convolutional neural network (ESCNN) and logistic regression (Table 4) [10,11,12,13,14,15,16,17,18,27]. Our results are like those published by Gonçalves et al. [9], who used the DeepHP dataset and found high performance with VGG16 (AUC: 0.998), InceptionV3, and ResNet50 (AUC: 0.994). Similarly, Klein et al. [10] and Liscia et al. [11] reported high sensitivity and specificity using VGG-style networks on Giemsa- and silver-stained slides, respectively, though variability in precision was noted due to staining technique and patch content. Martin et al. [12]. demonstrated robust performance using HALO-AI, with accuracy exceeding 98% across multiple metrics. Other studies, such as Ibrahim et al. [13] and Zhou et al. [14], confirmed the utility of ResNet and DenseNet variants in HP detection, with AUC values above 0.93 and F1 scores nearing 89%. Krishna et al. [15] recently reported superior performance using the BoostedNet model (F1 score: 98.31%). Unlike these, our results demonstrated a different performance trend with the BoostedNet model (Table 3).

In addition, findings in this work evidenced that ML models can outperform traditional diagnostic methods. For instance, the recall and specificity reported for H&E staining in the detection of HP reach maximum values of 93% and 90%, respectively [28]. In contrast, our results showed that the InceptionV3 model can achieve up to 100% recall and 94% specificity (Table 3).

AutoML streamlines the development of machine learning models by handling essential processes such as data preparation, feature selection, model design, and hyperparameter tuning. This automation has made AutoML an attractive solution in biomedical imaging, especially for users without extensive expertise in deep learning. AutoKeras, an open-source framework, stands out for its ease of use and adaptability across a variety of applications [29]. Recent studies, such as the work by Elangovan et al. [30], have benchmarked AutoKeras against bespoke DL models across diverse medical imaging datasets. Their findings suggest that AutoKeras can, in some cases, outperform manually crafted models, albeit at the cost of significantly longer training times. Furthermore, their study revealed that increasing the number of trials or using higher-resolution images does not always correlate with better performance, challenging common assumptions in AutoML configuration.

In our study, we build upon this evidence by evaluating AutoKeras for the classification of HP infection in gastric biopsy samples—a task that has not previously been explored using AutoML frameworks, to our knowledge. While AutoKeras facilitated a rapid and accessible modeling workflow, it underperformed in comparison to traditional DCNN models, such as VGG16, ResNet50, and InceptionV3, which were manually fine-tuned for our specific histopathological task. These results suggest that while AutoML platforms hold substantial promises for democratizing AI in medical imaging, they may still fall short in scenarios requiring domain-specific feature sensitivity and interpretability. Nonetheless, AutoML remains a valuable tool for early-stage model development, rapid prototyping, and expanding access to AI methods in resource-limited or low-code environments.

Regarding the data acquisition process, one of the notable strengths in the present article is the use of IHC prior to annotating the H&E slides, as well as the detailed annotation of glandular and inflammatory cells in proximity to HP. The identification of HP may have some limitations in WSIs. Recently, Chen, et al. [31] found that the diagnostic accuracy using light microscopy versus digital slides based solely on H&E staining was 81% and 72%, respectively, a statistically significant difference (*p* = 0.0142). When HP IHC slides were provided, the diagnostic accuracy improved to comparable rates (96% light vs. 99% digital, *p*: 0.2199). Due to these limitations, they recommend reviewing glass slides and/or performing ancillary stains, especially when there is a discrepancy between the degree of inflammation and the presence of microorganisms on digital images.

## 5. Conclusions

In conclusion, the findings underscore the strong viability of transfer learning with established CNN architectures for histopathological image classification. Pretrained models such as InceptionV3, VGG16, and ResNet50 achieved high accuracy in distinguishing gastric biopsies with and without HP infection. In contrast, automated machine learning approaches like AutoKeras exhibited notably lower performance. These results reinforce the advantage of leveraging deep pretrained networks, which provide robust and generalizable feature representations for medical image analysis tasks. Our findings support the potential of DL in gastric pathology, while also emphasizing the need for improved annotation granularity and enhanced training strategies to address morphological heterogeneity in future work.

It is important to recognize that no single ML model universally excels across all pathology applications. Each context presents distinct histological patterns, staining variability, and imaging conditions that may impact model performance. Therefore, we advocate for a problem-specific modeling strategy, whereby the selection and evaluation of DL architectures are driven by the nuances of the target task.

In addition, given that WSIs have become increasingly prevalent in pathology practices worldwide, and considering current limitations in detecting HP in WSIs, we recommend employing DL models for automated classification within computational pathology. This approach can enhance diagnostic accuracy, reduce observer variability, and significantly decrease diagnostic turnaround time.

## Figures and Tables

**Table 1:** Distribution of patients, biopsies, and image patches across training and test sets.

Phase	Patients	Biopsies	Patches Included	Notes
Training	16 (10 HP+/6HP−)	80 (48 HP+/32HP−)	Yes	Used for model fitting and augmentation
Test	4 (2 HP+/2HP−)	20 (12 HP+/8HP−)	Yes	Held-out internal test set

**Table 2:** An overview of the evaluation training metric results obtained for the machine learning models.

DL Model	Accuracy	Precision	Recall	Specificity	F1 Score	MCC
InceptionV3	98%	97%	100%	97%	98%	97%
VGG16	98%	97%	100%	97%	98%	96%
ResNet50	97%	97%	99%	96%	98%	95%
BoostedNet	85%	87%	84%	87%	86%	82%
AutoKeras	89%	92%	85%	93%	88%	78%

**Table 3:** An overview of the evaluation test metric results obtained for the machine learning models.

DL Model	Accuracy	Precision	Recall	Specificity	F1 Score	MCC
InceptionV3	97%	94%	100%	94%	97%	93%
VGG16	96%	94%	98%	94%	96%	92%
ResNet50	96%	94%	97%	94%	96%	91%
BoostedNet	83%	84%	83%	84%	84%	68%
AutoKeras	82%	85%	80%	84%	82%	64%

**Table 4:** Characteristics of the deep learning models previously reported in the scientific literature.

Authors and Year (Ref)	Databases	Validation Stain	AUC (IC 95%)	DL Architecture	Additional Pre-Processing	xAI	Metadata	Total Number of WSIs	Patch Size (Pixel)	Training Set (WSIs)	Validation Set (WSIs)	Test Set (WSIs)	External Validation
Present article	Institutional	H&E	1.0 (N/A)	InceptionV3	Quality filtering: patches containing more than 60% background or minimal/no visible tissue were discarded. Data augmentation (rotation, horizontal flip, zoom, and width and height shift)	No	Yes	100	512 × 512	80	N/A	20	Yes
			1.0 (N/A)	VGG16									
			1.0 (N/A)	ResNet50									
			0.92 (N/A)	BoostedNet									
			0.92 (N/A)	AutoKeras									
Cano, et al., 2025 [17]	Institutional	IHC	0.961 (N/A)	Autoencoder	Morphological operations, conversion to HSV and pixel filtering, sliding windows on the edges	No	No	245	256 × 256	123	N/A	122	No
			0.77 (N/A)	ResNet18									
			0.92 (N/A)	ResTreshold									
			0.88 (N/A)	UNI Vit									
Krishna, et al., 2024 [15]	Public dataset	H&E, Giemsa	0.990 (N/A)	BoostedNet: CNN + XGBoost	Resize to 256 × 256, Gaussian filter, data augmentation (rotation, zoom, shear, flip)	Yes	No	19	256 × 256	N/A	N/A	N/A	Yes
Ibrahim, et al., 2024 [13]	Institutional	H&E	0.941 (N/A)	ResNet-101	N/A	No	No	204	960 × 1280	CV	CV	CV	No
			0.930 (N/A)	DenseNet-201									
			0.903 (N/A)	MobileNet-v2									
			0.917 (N/A)	InceptionV3									
			0.907 (N/A)	Xception									
Lin, et al., 2023 [16]	Institutional	H&E	0.973 (0.954–0.993)	ESCNN + Logistic Regression	N/A	Yes	No	1075	N/A	885	N/A	190	Yes
Franklin, et al., 2022 [27]	Institutional	H&E	N/A	HALO-AI software (fully convolutional VGG network)	Data augmentation: rotations variations in hue, saturation, contrast, and brightness	No	Yes	187	400 × 400	112	N/A	75	No
Liscia, et al., 2022 [11]	Institutional	W-S	0.938 (N/A)	CNN-based model via Microsoft Custom Vision (VGG-based model)	NDPI to TIFF conversion	No	Yes	185	2000 × 2000	N/A	N/A	N/A	No
Gonçalves, et al., 2022 [9]	Institutional	H&E	0.998	VGG16	Noise correction, grayscale, binarization, augmentation (rotation, flip, zoom)	No	No	19	256 × 256	N/A	N/A	N/A	No
			0.994	InceptionV3									
			0.994	ResNet50									
Martin, et al., 2020 [12]	Institutional	H&E	1.00 (N/A)	HALO-AI software (fully convolutional VGG network)	Data augmentation with random rotations and random changes in hue, saturation, contrast, and brightness	No	Si	300	400 × 400	210	90	N/A	Yes
Klein, et al., 2020 [10]	Institutional	Giemsa	0.950 (N/A)	Compact VGG-style deep neural network	Data augmentation, Rgb to Hsv conversion, Otsu’s thresholding, morphological operations, contour detection	Yes	Yes	627	224 × 224	477	150	N/A	Yes
			0.902 (N/A)										
			0.810 (N/A)										
Zhou, et al., 2020 [14]	Institutional	H&E	0.965 (0.934–0.987)	MobileNet-V2	Laplacian filtering, data augmentation through horizontal inversion	Yes	Yes	108	299 × 299	77	31	N/A	No

CV: Cross Validation. xAI: Explainable Artificial Intelligence. WSI: Whole-Slide Image. N/A: Not Applicable. IC: Confidence Interval. AUC: Area Under The Curve. DL: Deep Learning. Institutional: Institution’s own database. W-S: Warthin–Starry stain.

## Data Availability

The original contributions presented in this study are included in the article. Further inquiries can be directed to the corresponding author.
